# Airway Natural Killer Cells and Bacteria in Health and Disease

**DOI:** 10.3389/fimmu.2020.585048

**Published:** 2020-09-25

**Authors:** Maud Theresine, Neha D. Patil, Jacques Zimmer

**Affiliations:** CG I Group, Department of Infection and Immunity, Luxembourg Institute of Health, Esch-sur-Alzette, Luxembourg

**Keywords:** natural killer cells, bacteria, infection, lungs, airways, chronic obstructive pulmonary disease, pathogenesis

## Abstract

Natural killer (NK) cells are innate lymphoid cells at the interface between innate and adaptive immunity and mostly studied for their important roles in viral infections and malignant tumors. They can kill diseased cells and produce cytokines and chemokines, thereby shaping the adaptive immune response. Nowadays, NK cells are considered as a strong weapon for cancer immunotherapy and can for example be transduced to express tumor-specific chimeric antigen receptors or harnessed with therapeutic antibodies such as the so-called NK engagers. Whereas a large body of literature exists about the antiviral and antitumoral properties of NK cells, their potential role in bacterial infections is not that well delineated. Furthermore, NK cells are much more heterogeneous than previously thought and have tissue-characteristic features and phenotypes. This review gives an overview of airway NK cells and their position within the immunological army dressed against bacterial infections in the upper and predominantly the lower respiratory tracts. Whereas it appears that in several infections, NK cells play a non-redundant and protective role, they can likewise act as rather detrimental. The use of mouse models and the difficulty of access to human airway tissues for ethical reasons might partly explain the divergent results. However, new methods are appearing that are likely to reduce the heterogeneity between studies and to give a more coherent picture in this field.

## Introduction

Historically, human natural killer (NK) cells have mostly been harvested from and studied in peripheral blood (PB), which is an easy way to access them, and where they usually represent 5–20% of all lymphocytes (1–3). Two different subsets have been initially described, called CD56^bright^CD16^–^ (up to 10% of PB NK cells) and CD56^dim^CD16^bright^ (at least 90% of PB NK cells). Phenotypic and functional (cytokine production for the former and cytotoxic activity for the latter) characteristics distinguish both populations (1–3). However, things are not that simple, as four additional subpopulations have been identified, which are (i) CD56^bright^CD16^dim^, (ii) CD56^dim^CD16^–^, (iii) CD56^–^CD16^bright^ and finally (iv) CD56^dim^CD16^dim^ (4), the latter still being almost systematically overlooked in the literature (5). Human NK cell functions are governed by a balance between the messages transmitted through inhibitory receptors (IR), such as KIR, CD94/NKG2A, ILT2, TIGIT, and activating receptors (AR), such as particularly NKG2D and the natural cytotoxicity receptors (NCR) NKp46, NKp30, and NKp44 (6). When stimulated, NK cells exert natural cytotoxic activity against tumor cells and virally infected cells, antibody-dependent cellular cytotoxicity (ADCC) toward antibody-coated target cells via the Fcγ receptor CD16, and cytokine and growth factor production (2, 6).

Most of the ligands of the IR are represented by Human Leukocyte Antigen (HLA) class I molecules, so that target cells lacking those molecules in part or in total, become killed by the NK cells. The IR nevertheless have another important function, as they are responsible for NK cell education. Indeed, before a developing NK cells becomes functional, its self-specific IR must interact with their ligands expressed by cells in their micro-environment (7, 8). NK cells without such IR, which can represent up to 20% of all PB NK cells, remain uneducated, and hyporesponsive (7, 8). However, they can be activated under certain conditions, such as some viral infections (9).

A hot topic in the NK cell field is of course their potential use as immunotherapeutic anticancer agents. To reach this aim, several approaches are studied, and for example the chimeric antigen receptor (CAR) NK cells, which allow the specific targeting of a tumor antigen (10), or the use of multi-specific antibody constructs directed simultaneously at several NK cell AR and tumor surface molecules (6), appear as particularly promising. It has also been discovered that NK cells, which had been previously considered as exclusively innate immune cells, can develop a memory-like behavior (11). Finally, NK cell metabolism, which appears to be different between educated and uneducated cells, is extensively studied (12, 13).

Another aspect that has changed our view on NK cells in recent years is the observation of a broad heterogeneity of this population. Not only are there many subsets in PB based on the clonal distribution of several IR, immature, partly mature and completely mature fractions based on the relative expression of CD56, CD16 and the IR NKG2A and KIR (14), conventional and adaptive NK cells (14, 15), but there are also heterogeneous aspects between PB and various tissues (15, 16). Very recent data by Dogra et al. (17) suggests a model in which the phenotype, the degree of maturity and the functions of NK cells are closely dependent on the anatomic location, with no influence of age and gender.

## NK Cells in the Upper Airways

It is quite difficult to find a substantial amount of references regarding upper airway NK cells. In human, the articles were mostly reporting on the investigation of NK cells in chronic rhinosinusitis, an inflammatory state of the mucosa of the nose and the sinuses (18) with a significant impact on quality of life. Two different forms, one with nasal polyps and one without nasal polyps, are distinguished (19, 20). Bacterial pathogens are considered as one of the etiological factors in this disease (18). However, as the bacteriology of ethmoidal biopsies was the same regardless of the presence or absence of polyps, Niederfuhr et al. questioned the bacterial role in the pathogenesis of the polyps as well as a systematic antibiotic treatment (19). In a study of 18 patients, further subdivided into those responding and those resistant to treatment, and 19 healthy controls, Kim et al. investigated exclusively PB NK cells. The authors demonstrated that the PB NK cells from the patients had decreased effector functions compared to the healthy controls, with the treatment-resistant individuals being most severely affected (18). The recent manuscript by Kaczmarek et al. (20) reported not only on PB NK cells, but also on those from nasal mucosa and from nasal polyps. However, the exploitation of the material was limited to CD3^–^CD56^+^CD16^+^ events, which excluded the population of CD56^bright^CD16^–^ NK cells that might be numerically well represented in these tissues. The phenotypic investigations of this subset in the nose revealed a predominance of relatively immature, CD27^+^ NK cells. Furthermore, the AR NKG2D was expressed at lower frequencies (lower percentages of NKG2D^+^ cells) and lower density of expression in the nasal mucosa and the polyps compared to PB (populations negative for NKG2D were identified in the tissues). Finally, the percentage of NK cells among lymphocytes (mean: 33%) was significantly higher in the polyps than in PB (20).

Okada et al. published a paper about NK cells in the nasal mucosa of the mouse on the C57BL/6 background (21), in which they showed that these NK cells were more immature (according to the relative levels of expression of CD27 and CD11b) and phenotypically more activated (reduced expression of CD62L, higher percentage of CD69^+^ cells) than those from spleen and lung. Around 12% expressed the tissue residency and activation marker CD69 and one third of those also CD103. The pattern of expression of the Ly49 receptor family was different between the three tissues. Functionally [CD107a staining and interferon (IFN)-γ production], nasal NK cells appeared to be hyporesponsive compared to their spleen, but not their lung counterparts (21), which might be related to the possibility that the fraction of CD69^+^ NK cells was not sufficient to significantly activate the global NK cell population in the chosen experimental readouts.

Although this dataset is interesting *per se*, it should not be ignored that Casadei and Salinas (22), in a review about different animal models of nasal infections and immunity, cited several anatomic (functional vomeronasal organ in contrast to human, no Waldeyer’s ring) and physiologic (macrosmatic, no coughing-sneezing reflex, lower sensitivity to human viruses) limitations of the mouse in this context, so that such results should always be considered with care before extrapolating to the human situation.

## NK Cells in the Lungs

Lung NK cells have recently been extensively reviewed in this journal (23, 24), so that a summary of their most important features might be sufficient. Lungs are constantly exposed to microparticles from the environment. Particularly, as the mucosal lung epithelium is at the interface between the outside world and the organism, it can become the entry site for infectious pathogens, be they bacterial, fungal, or viral in nature. Therefore, an extensive and sophisticated local immune response is waiting to be triggered at this anatomic location, and human NK cells, which represent around 5–20% of lung lymphocytes (24), are a part of it. The work of Marquardt et al. has established that most human lung NK cells represented the circulating subset and had the mature CD56^dim^CD16^bright^ phenotype (25). They expressed more frequently the differentiation marker CD57 as well as educating KIR than blood NK cells from the same donors but were relatively hyporesponsive upon stimulation with HLA class I-negative target cell lines. In addition, however, a putative tissue-resident subset (around 20% of all lung NK cells), further subdivided into relatively immature CD56^bright^CD16^–^ and CD56^dim^CD16^–^ cells (24, 25), expressed the tissue residency markers CD69, CD49a, and CD103. These cells were characterized in detail again by Marquardt et al. (26), who showed that they were functional, especially after stimulation with the cytokine interleukin (IL)-15 and displayed a unique transcriptional profile. Several subpopulations could be distinguished based on the relative expression of CD49a and CD103 (24, 26).

Natural killer cells have likewise been investigated in mouse lungs, particularly by Wang et al. (27) and Michel et al. (28). Both groups found that lung NK cells were more mature than those from the spleen (28) or other organs (27) according to the relative expression of CD27 and CD11b. Whereas the former authors described a higher expression level of the IR CD94/NKG2A and a lower level of the AR NKG2D, the second paper could confirm this data only regarding NKG2D in terms of mean fluorescence intensities. Lung NK cells proliferated less, degranulated less (CD107a assay) and were less cytotoxic than splenic NK cells (28), but these functions were rapidly up-regulated upon bacterial lung infection (27). This suggests that at homeostasis, lung NK cells are inhibited to avoid damage to normal autologous cells, but that they can quickly intervene in case of an infectious insult (27). Michel et al. showed in *in vitro* co-culture systems that both spleen and lung macrophages could significantly up-regulate the cytotoxic activity of lung NK cells through a contact-dependent mechanism (28).

Regarding the homeostatic situation, research in recent years has revealed that in contrast to the older view of the lungs as sterile organs, a lung microbiota is present in the lower airways which exerts significant effects in health and disease, although it is not as abundant as in the gut (29–32). The term “microbiota” refers to all the microorganisms present, namely bacteria, fungi, protozoans, and viruses (29), but here we will only consider the role of bacteria. Six phyla are predominantly represented in the lower airways: Firmicutes, Proteobacteria, Bacteroidetes, Actinobacteria (31, 32), Acidobacteria, and Fusobacteria (32). This microbiota is supposed to be transient in healthy donors and to be established from micro-aspiration and inhalation (32) and its composition at any given time point submitted to the parameters of bacterial arrival, bacterial removal, and local immune responses (32, 33). In this way, an equilibrium state is reached that depends also strongly on the gut microbiota through various bacterial metabolites and contributes to the maintenance of homeostasis in the lower airways (gut – lung axis) (32–34). Everything that disturbs this balance, such as some medications and particularly antibiotics, increases in nutrients (high fat diet, low fiber diet), cigarette smoke, infectious agents, chronic inflammation, can disturb the gut as well as the lung microbiota and lead to a state of dysbiosis, characterized by an increased number of airway bacteria and a change in its composition. The dysbiosis is profoundly linked to several severe lung diseases [asthma, chronic obstructive pulmonary disease (COPD), infections, cancer] (29–35).

Natural killer cells have, to our knowledge at least, not been investigated in detail in the context of a normal lung microbiota to date. As most lung NK cells are not activated nor tissue-resident (as illustrated by their negativity for CD69), they might not react very strongly to a normal microbiota. However, as they are expressing several bacteria-specific toll-like receptors (TLRs) that signal in the presence of bacterial pathogens (36), it might be conceivable that they could also mount an immune response toward microbiota components and that this would contribute to homeostasis. The overall immunosuppressed state of lung NK cells at baseline would help to avoid aggression of harmless and useful bacteria and of uninfected autologous cells (31). Yang et al. (31), as well as Fuchs and Colonna (37), discuss data claiming that at steady state, alveolar macrophages secrete immunosuppressive cytokines which keep NK cells in respect. This is in contrast with the results of Michel et al. (28), discussed above. However, the macrophages and dendritic cells (DC) switch to pro-inflammatory cytokine production in case of a bacterial or viral infection and thereby activate the NK cells.

## Chronic Obstructive Pulmonary Disease

This entity is the third cause of mortality in the United States of America (3) and worldwide (38) and is in most cases the consequence of prolonged cigarette smoking (39). It is characterized by airflow obstruction, emphysema, recurrent infections (24, 39), chronic inflammation, and overproduction of mucus (40). Acute exacerbations significantly limit the quality of life of the patients (38, 39). The exacerbations are in principle caused by viral or bacterial infections, the latter most frequently due to *Haemophilus influenzae*, *Streptococcus pneumoniae*, and *Moraxella catarrhalis* (39). *Pseudomonas aeruginosa* is another bacterium frequently involved and one of the most dangerous ones, based on its highly pathogenic properties (39), and its remarkable level of resistance to antibiotics.

Natural killer cells have been investigated in human COPD as well as in animal models of this disease. Motz et al. demonstrated that exposure of pulmonary leukocytes to viral pathogen-associated molecular patterns (PAMP) induced higher functional properties (degranulation measured with the CD107a assay, and IFN-γ production) *ex vivo* in chronic cigarette smoke exposed than in non-exposed C57BL/6 mice (40). Interestingly, bacterial PAMP appeared to be less efficient in this model, as among five molecules tested, only FSL-1 (bacterial lipopeptide, TLR2/6 agonist) and lipopolysaccharide (LPS, TLR4 ligand) increased the percentage of IFN-γ^+^ NK cells above the one of the non-exposed mice. In contrast, other papers reported that NK cell functions are diminished in COPD (41).

It has been further repeatedly demonstrated that in COPD or relevant animal models, NK cell cytotoxic activity is increased relative to non-COPD smokers and healthy individuals (23, 24). Based on the model of lung NK cell hypo-responsiveness at baseline, cigarette smoke and even more the inflammatory state of the lower airways in COPD would activate the alveolar macrophages and induce their production of pro-inflammatory cytokines. These would, in turn, unleash the NK cells and increase their cytotoxic activity, cytokine and chemokine expression, leading to a further aggravation of the inflammation and the clinical status of the patients.

Indeed, in accordance with this concept, Freeman et al. (42) showed that CD56^+^ cells (in fact a mixture of NK cells and CD56^+^ T lymphocytes) isolated from lung parenchymal samples of non-COPD smokers and COPD patients with a smoking history, although similar in terms of frequencies between the cohorts, had a different cytotoxic activity toward autologous lung epithelial cells. The CD56^+^ lymphocytes from the COPD patients were more cytotoxic than the cells from the non-COPD smokers, in an experimental setup without additional stimulation. The target cells were supposed to be mostly epithelial cells based on their positivity for CD326, their size, and their negativity for the hematopoietic cell marker CD45. The cytotoxicity was measured as the percentage of Annexin V^+^ target cells after the co-culture with the effectors and was around 10% in most samples. This was not a lot, but the NK cells and CD56^+^ T cells were not otherwise activated. Most of the parenchymal lung NK cells were CD56^+^CD16^+^ and the minor rest CD56^+^CD16^–^ (42).

Another study was provided by the same group in 2018 (43). It showed that isolated, purified lung NK cells induced apoptosis in autologous epithelial cells. This time, the mean level of cytotoxicity was rather high compared to the previous paper, and it was very significantly stronger in COPD patients than in non-COPD smokers. The NK cells, but not the target cells, determined this increased cytotoxic activity, because K562, a HLA class I-negative myeloid leukemia cell line used as the standard NK cell target, was also lysed more efficiently by COPD NK cells than by their non-COPD counterparts. The authors confirmed their data in a mouse model and then showed that the NK cells were primed by DC *via trans*-presentation of IL-15 (43), a phenomenon first described in 2007 by Andreas Diefenbach and his group (44). This would nicely explain the higher level of NK cell cytotoxicity observed in COPD.

Along the same line, Okamoto et al. administered IL-2 and IL-18 to normal mice and observed a lethal effect within 4 days, selectively involving the lungs, with a profound interstitial infiltration of lymphocytes dominated by NK cells (45). High levels of various cytokines and chemokines were found in serum and lungs. Depletion of the NK cells by antibodies completely abrogated the lethal injury, which is a convincing demonstration of the potentially destructive power of NK cell-activating cytokines and NK cells themselves (45). This work was intended as a contribution to the elucidation of the pathogenesis of interstitial pneumonia, but similar mechanisms, in the presence of high levels of pro-inflammatory cytokines in bacterial infections, might contribute to COPD. In human cancer patients, administration of high dose IL-2 induced a vascular leakage syndrome where the so-called lymphokine activated killer cells (equivalent to highly activated NK cells) destroyed endothelial cells, causing a generalized edema, and damaging several organs (46).

Hodge et al. demonstrated a higher number of NK cells in the bronchoalveolar lavage fluid (BALF) of COPD patients (the cohort was composed of current smokers and of ex-smokers) than in healthy smokers (47), a higher content of the cytolytic molecule granzyme B and, most importantly, a significantly increased cytotoxic activity against K562. They also found a reduction in the percentage of BALF NK cells expressing CD94 (which they consider as IR, although it is more a chaperone molecule for the true IR NKG2A). Nevertheless, this indirect measure of a down-regulation of NKG2A could indicate that it contributes to the higher NK cell cytotoxic activity observed in COPD (47).

Recently, Osterburg et al. presented a multiparameter flow cytometry study of PB NK cells from COPD patients compared with smokers and never smokers (38). In contrast to those, COPD patients and smokers highly expressed the maturation marker CD57 as well as the AR NKp46 and NKp44 (normally only present on activated but not on baseline NK cells), but lower levels of CD56. Certain NK cell subpopulations were indicative of prior exacerbations (38).

The AR NKG2C, which is significantly present only on adaptive NK cells from human cytomegalovirus (CMV)-infected individuals, was not differentially expressed in PB of COPD patients with a smoking history and healthy volunteers, but present on a higher percentage of NK cells in the patients with the most frequent exacerbations and the most reduced lean mass (48). A relationship with the bacterial burden cannot be excluded in this context, as there might be a correlation between the viral reactivations and the bacterial colonization, contributing together to the higher number of exacerbations.

Most of the papers discussed above investigated the NK cell cytotoxicity toward autologous cells or conventional NK target cells, but what about a potential direct bacterial killing? NK cells, upon appropriate stimulation, release cytolytic molecules called perforin, granzymes and, in human but not in mice, granulysin, which have an additive or synergistic cytolytic effect toward bacteria (49). They can form pores in the target cell walls and thereby eliminate the microorganisms, but in addition they are able to eliminate some types of eukaryote cells infected by bacteria (41, 49, 50). Furthermore, in addition to the direct effect, NK cells are embedded in the immunological network and react (through an increased cytotoxic activity and cytokine production) to the immune cells and the cytokines/chemokines in their environment (50), which is strongly shaped in case of a bacterial infection [“cellular crosstalk” (50)].

Data about chronic rhinosinusitis, nasal polyposis and COPD are summarized in Table 1.

**TABLE 1:** Natural Killer (NK) cells in airway diseases.

Disease	Species	Origin of NK cells	Effect on NK cells	References
Chronic rhinosinusitis	Human	Peripheral blood	↓ Effector functions	(18)
Nasal polyps	Human	Nasal mucosa	↓ NKG2D; ↑ CD27	(20)
COPD model	Mouse	Lung	↑ IFN-γ	(40)
COPD	Human	Lung	↑ Cytotoxicity	(43)
COPD	Human	BALF	↑ Cytotoxicity	(47)
			↑ Number	(47)
COPD	Human	Peripheral blood	↑ CD57, NKp46, NKp44	(38)

*Consequences of the indicated diseases on NK cell phenotype and functions. COPD, chronic obstructive pulmonary disease; BALF, bronchoalveolar lavage fluid; and IFN-γ, interferon-gamma.*

### Pseudomonas aeruginosa

As mentioned above, this ubiquitous Gram-negative pathogen is part of those colonizing the lower airways in COPD, but it is also a major problem in cystic fibrosis and in nosocomial infections, with a high morbidity and mortality (51). The role of NK cells in the host defense against this bacterium has been quite extensively studied by the team of Michael T. Borchers (51, 52) in mouse models. In the chronologically first work, outbred CD-1 mice were intranasally infected with the *P. aeruginosa* laboratory strain PAO1 (52) and evaluated 24 h later. The findings can be summarized as follows: (i) the infection increased the expression of ligands for the AR NKG2D, present on almost all NK cells but also on a subpopulation of CD8^+^ T lymphocytes, *in vivo*; (ii) similarly, these ligands increased in an infected human lung epithelial cell line *in vitro*; (iii) the inhibition of the AR NKG2D with a monoclonal antibody significantly reduced the clearance of *P. aeruginosa* from the lungs; (iv) antibody-mediated NKG2D blockade down-regulated the amount of the cytokines IL-1β, IFN-γ and tumor necrosis factor (TNF)-α and in addition of nitric oxide; and finally, (v) the same experiment also revealed a threefold reduction of epithelial cell damage, measured as shedding of these cells into the BALF (52). The latter point brings us again to the recurrent theme of lung cell damage that can be induced by activated NK cells, whereby it would have to be determined if this is beneficial (elimination of infected cells by NK cells) or deleterious (exaggerated damage to the epithelium).

The follow-up paper (51) then presented a conditional mouse model with an inducible expression of NKG2D ligands on lung epithelial cells. Here, the bacterial clearance was significantly higher in those mice that overexpressed the NKG2D ligands. Moreover, the survival up to 96 h post-infection and the level of phagocytosis were significantly increased in the latter group. Similarly, in *in vitro* experiments, where the NK cells were stimulated with LPS, the percentage of NK cells producing IFN-γ was much higher in the mice with the increased expression of NKG2D ligands. As expected, this percentage dropped (but was not completely abolished) in NK cells from infected mice treated with an anti-NKG2D antibody (51).

However, the *P. aeruginosa*-derived exotoxin A, which in combination with IL-1α may induce a dangerous inflammatory state with tissue damage in the host, has also been shown to inhibit NK cell cytotoxic activity against K562, even in the presence of usually stimulating cytokines such as IL-2 (53). The inhibition was almost complete with a high dose of the toxin and still partial with a low dose (53). The effector cells were not purified NK cells but peripheral blood mononuclear cells (PBMC), so that an indirect effect on the NK lymphocytes might play a role in this readout.

Furthermore, Pedersen and Kharamzi described already in 1987 that the *P. aeruginosa*-derived alkaline protease and elastase inhibited NK cell cytotoxic activity against K562, presumably due to a reduction in the effector-target conjugate formation (54). In addition, these molecules strongly reduced the binding of an anti-CD16 (called Leu-11 at that time) antibody (54).

### *Burkholderia cepacia* Complex

This group of pathogenic Gram-negative bacteria is composed of several species, of whom some are dangerous for cystic fibrosis patients, as they are highly resistant to multiple antibiotics (55). Li et al. (55) investigated the interaction between *Burkholderia cenocepacia* and NK cells, and first demonstrated that the NK-like leukemia cell line YT (56), as well as primary purified human NK cells, significantly reduced the number of living bacteria (measured as CFU) after a co-incubation of 2 to 4 h. The results were confirmed with life cell imaging techniques and bacterial uptake of propidium iodide (PI). The authors then wanted to know if the killing activity was contact-dependent or not, and first showed that YT cells bound the fluorochrome-labeled bacteria. Then, they could demonstrate that a direct contact was needed for the killing activity, as nothing happened to the bacteria when they were separated from the NK cells by a porous membrane, allowing passage of soluble molecules but not of cells (55). Most bacteria remained extracellular and were not taken up by the YT cells. Killing was almost completely abrogated after treatment with strontium chloride (SrCl_2_), which is known to deplete NK cells from their cytotoxic granules (57). Finally, it was established that Src family kinases were activated in YT cells after the contact with *B. cenocepacia* (55). This is a very nice demonstration that NK cells are able to directly kill certain extracellular bacterial species through NK cell – bacteria contact, although the precise mechanism is still unknown. Other possible mechanisms of NK cell-mediated elimination of bacteria are the lysis of intracellular pathogens within the infected cells and the activation of other immune cells, and particularly of macrophages, via NK cell-derived cytokines (such as IFN-γ) (55), and most likely also the killing of bacteria-infected cells expressing ligands for NK cell AR.

### Klebsiella pneumoniae

This is another Gram-negative pathogen which poses a major problem due to its frequent causative involvement in nosocomial infections (particularly in pneumonia) and the steady increase of strains multi-resistant to antibiotics (58). Chalifour et al. (59) demonstrated that the outer membrane protein A (KpOmpA) from this microorganism, known to signal via TLR2, induced IFN-γ and α-defensin (an antimicrobial peptide) synthesis and release in human NK cells. In the mouse, both NK cell-derived IFN-γ (58) and IL-22 (60) have been described to be necessary for bacterial clearance (58, 60). In the paper from Xu et al., it was nicely shown with genetic controls and depletion experiments that the immune defense against this pathogen indeed deeply involved NK cells and that a subset of them produced IL-22. The NK cells had a conventional and mature phenotype (less CD27^+^, more KLRG1^+^) distinct from other innate lymphoid cells (ILC) (60). Ivin et al. focused on the fact that IFN-γ production by NK cells, likewise necessary for the elimination of the bacteria through a network with alveolar macrophages, was dependent on the NK cell-intrinsic stimulation by type I IFN, in turn induced by *K. pneumoniae* (58). In contrast to the crucial role of NK cell-derived cytokines, their granzymes (A and B), one of the constituents of the lytic granules, did not seem to play a major role in this model (61). However, this does not rule out that in human, granulysin and perforin together might have a cytotoxic effect on these bacteria.

## Other Gram-Negative Bacteria

In the case of *Helicobacter pylori*, responsible for chronic gastric inflammation with the potential to lead to ulcers or cancer, pre-incubation with fixed bacteria increased the cytotoxic activity of NK cell-enriched PBMC toward K562 and other tumor target cells, as well as the release of IFN-γ (62). Furthermore, Rudnicka et al. showed that the bacterial glycine acid extract induced NK cell expansion and IFN-γ production, whereas the LPS from the same bacteria inhibited these parameters, and instead favorized the apparition of IL-10-producing NK cells (63). Although this might just marginally be relevant for NK cells in the lungs, it nevertheless shows to which extent these cells can react to bacteria and how the latter try to manipulate them.

*Legionella pneumophila*, the agent of Legionnaires’ disease, is replicating intracellularly in macrophages. Here again, NK cell production of IFN-γ, induced probably through direct TLR messages (64), IL-12 [produced by DC (65)], and IL-18 [produced by neutrophils (66)], was crucial for bacterial clearance from the lungs. In addition, Blanchard et al. had already observed in 1988 in a mouse model that this pathogen stimulated NK cells *in vivo* and *in vitro* to produce IFN-γ and to increase their cytotoxic activity to tumor cell lines, the highest levels having been measured in the lungs (67).

## Gram-Positive Bacteria

One of the most frequent culprits in community-acquired pneumonia is *S. pneumoniae*. Regarding the role of NK cells in this infection, their beneficial or detrimental action depended on the pathogen’s serotype (68). Thus, the control of serotype 1 depended on NK cells, as demonstrated by Baranek et al. in a mouse model (68). These authors investigated the consequences of a defect in the transcriptional cofactor Four-and-a-half LIM-only protein 2 (FHL-2) on NK cells in general and on pneumococcal infection particularly. It had been previously established that IFN-γ was, once more, the crucial factor in host defense in this context, and that NK cells were one of its major producers (69). In the spleen and the lungs of FHL-2 knockout (KO) mice, the number of NK cells and their expression of the AR NKG2D and NK1.1 (CD161c) were down-regulated and a negative effect of the deficiency on NK cell maturation was observed. Mortality to *S. pneumoniae* lung infection was strongly increased in the KO mice but could be rescued by the adoptive transfer of wildtype NK cells. Finally, the authors showed that IFN-γ production by NK cells was severely reduced and that less neutrophils were recruited to the lungs of the KO animals (68).

The role of the mostly immunosuppressive cytokine IL-10 in dampening the immune response to pneumococcal infection was shown in 1996, when van der Poll et al. administered the pathogen intranasally together with IL-10 and observed early mortality and reduced levels of the pro-inflammatory factors IFN-γ and TNF. Conversely, all this was restored when the mice were pre-treated with an anti-IL-10 antibody (70).

These results were very recently confirmed by Clark et al. (71), who worked with IL-10 reporter and IL-10-KO mice to observe that *S. pneumoniae* induced IL-10 production by NK cells (around 50% of total lung NK cells) with a negative effect on animal survival, and that the bacterial burden was diminished in the lungs of the KO mice compared to wildtype animals. NK cell depletion in the latter induced a strong reduction in the bacterial lung counts and in IL-10. Furthermore, IL-10-deficient mice had significantly more neutrophils and monocytes in the infected lungs. Finally, the virulence protein Spr1875 from *S. pneumoniae* was identified as the IL-10-inducing factor (71).

None of these papers investigated the potential balance between the pro-inflammatory and anti-inflammatory effects of IFN-γ and IL-10, respectively, on the outcome of this infection, which would anyhow have been technically challenging. One might suppose that IL-10 is there to down-regulate an overwhelming immune response that would damage lung tissues, but on the other hand, it might also be counterproductive to dampen it too much and thus to lose control over the pathogens (72). Other groups have described that human as well as mouse NK cells could produce and release IL-10 (73, 74), although, according to Perona-Wright et al., this only occurred in the case of a systemic, but not a localized, pulmonary infection (with the Gram-negative bacterium *Yersinia pestis*) (74). In the case of systemic infections with *Listeria monocytogenes* and *Y. pestis*, approximately 50% of blood NK cells became IL-10^+^, and the cytokine was produced by a NK cell subset circulating in blood prior to the infection (74).

Before studying *S. pneumoniae* (71), Clark et al. had already shown that *L. monocytogenes* elicits IL-10 production by NK cells via the virulence factor p60 (with, as a consequence, an inhibition of the recruitment and the activation of myeloid cells) in a mouse model of systemic infection, where the lungs were not further investigated (75).

Another frequently encountered nosocomial and multi-resistant infectious agent is *Staphylococcus aureus*. Small et al. could demonstrate the fundamental role of NK cells in the response to these bacteria in the case of mouse lung infections (76), as (i) NK cell numbers in the airways increased; (ii) *in vitro* contact with products from the pathogens activated NK cells; (iii) co-culture of NK cells with alveolar macrophages increased the phagocytic activity of the latter, (iv) IL-15-KO mice were much more susceptible to the infection than wildtype mice, whereas they had much more neutrophils and macrophages in the lungs; and (v) NK cell depletion rendered even wildtype mice highly sensitive, despite a conserved IL-15 production (76). These findings demonstrate indeed once again the important role of NK cells in immune defense against extracellular bacteria.

In accordance with this model, Zhao et al. showed that particular matter, associated epidemiologically with enhanced numbers of lung infections, diminished the amount of NK cells migrating to rat lungs in case of infection with *S. aureus*, whereas adoptive NK cell transfer restored a vigorous NK cell response (77). In *ex vivo* experiments, NK cells improved, as in the previous study, the phagocytosis of the pathogens by alveolar macrophages.

It is well known that after influenza, recovering patients are very susceptible to bacterial superinfection, notably by *S. pneumoniae* and *S. aureus* (78). The contribution of NK cells to this phenomenon was demonstrated in a mouse model of H1N1 influenza virus infection followed by intratracheal instillation of *S. aureus*. The sequentially double-infected mice were much more susceptible to the infection (weight loss, survival rate) than those receiving PBS or bacteria only. This went hand in hand with severe changes in the histopathological aspect of the lungs and a marked reduction of local NK cell numbers and TNF-α^+^ NK cells. Furthermore, the concentrations of TNF-α and of the chemokines IP-10 and MIP-1α were diminished in the BALF. Adoptive transfer of naive NK cells could restore the immune response. The NK cells needed TNF-α to perform their antibacterial effect and this was organized via an interaction with alveolar macrophages and increased phagocytosis (78).

The conclusion that might be drawn from all these papers is that NK cells are very important, at least in mouse models, for the immune response to and the defense against pulmonary infections due to Gram-positive bacteria, with, on the other hand, a detrimental influence of these lymphocytes in case they produce too much immunosuppressive factors [the same old story (72)].

### *Mycobacterium tuberculosis* and Other Mycobacteria

*Mycobacterium tuberculosis* is the agent of tuberculosis (TB), an infectious disease that puts a high burden on the populations in developing but also in developed countries and increasingly shows resistance to conventional antibiotics. It latently infects about 25% of the total population and becomes clinically apparent in ten million patients per year, according to estimations from the World Health Organization (WHO) (79, 80), rendering it a major public health issue. As recently reviewed by Cong and Wei (23), NK cells could interact with this intracellular pathogen through the AR NKp46, NKp44, and NKG2D, as well as TLR2. Although they became activated under these conditions, they seemed to play only a negligible protective role, according to Junqueira-Kipnis et al. (81). These authors showed in a mouse model that lung NK cells augmented in number within the first 3 weeks after exposure to aerosols containing the mycobacteria and up-regulated CD69, IFN-γ and perforin. However, their depletion did not at all change the kinetics of the infection. Human PB NK cells likewise up-regulate IFN-γ after contact with *M. tuberculosis* (23).

Barcelos et al. (82) compared PB NK cells in cohorts of patients with active TB, isolated tuberculin^+^ skin tests, and tuberculin^–^ healthy donors. They found a different subset distribution according to the cohorts, with putative TB-exposed but-resistant individuals (defined as those with a positive tuberculin test) having overall less NK cells but an increased percentage of CD56^–^CD16^+^, CD56^+^CD16^–^ and especially CD56^bright^CD16^–/+^ NK cell subsets compared to the other two donor groups. In contrast, TB patients displayed lower frequencies of CD56^+^CD16^+^ cells. The authors speculated that this different subset distribution might have been related to the resistance or sensitivity to active TB, but of course, as the cells stem from PB, this dataset might have to be interpreted with some caution.

Surprisingly, however, Roy Chowdhury et al. (80), by following a cohort of adolescents from an endemic region in South Africa, could demonstrate with mass cytometry and functional experiments, that latent TB was associated with increased responses of PB NK cells, with a particular role for the AR CD16. Indeed, the percentages of NK cells among total living cells, of total CD16^+^ cells, of granzyme B^+^ and of perforin^+^ cells were significantly higher in patients with latent TB than in healthy, non-infected donors. In addition, ADCC (mediated via CD16) against P815 target cells was also higher in latent TB. By following further cohorts, the authors found that the percentage of PB NK cells was dynamically regulated during latency, progression of the disease and responses to antibiotic medication. This level of NK cells in PB even correlated inversely with inflammation in the lungs of patients with active TB (80). Such observations push NK cells again at the forefront of immune defenses in TB and at a possible role in the maintenance of latency.

With a similar cohort-based approach, Harris et al. (79) evaluated NK cell phenotype and functions in individuals with latent TB compared with healthy controls. Furthermore, participants were separated in infected and non-infected in a TB-endemic region in Kenya and a healthy volunteer cohort from the United States. Among the three groups, the persons from the United States had the significantly lowest percentage of CD56^–^ cells, which are known to expand in chronic human immunodeficiency virus (HIV) infection and other viral diseases and to be dysfunctional (83). The Kenyan volunteers displayed, among CD56^dim^ NK cells, a higher expression of granzyme B and of the non-MHC class I-specific IR TIGIT, with the highest levels found in the healthy cohort. Furthermore, these individuals had an increased expression of the AR NKp46. Within the CD56^bright^ subpopulation, the Kenyan participants showed increased expression of NKG2D but again decreased levels of NKp46, compared to the cohort from the United States. Functionally, degranulation (CD107a assay), IFN-γ production (intracellular flow cytometry) and CD69 expression were compared between the three cohorts after co-culture with K562 cells (evaluation of natural cytotoxicity) and P815 mouse cells plus anti-mouse antibody (evaluation of ADCC). Whereas for the latter parameter, no significant differences were observed, frequencies of CD69^+^, CD107a^+^, and IFN-γ^+^ NK cells turned out to be significantly higher in the United States study population, such as if an environment endemic for TB would impact the “missing self”-recognition capacities of NK cells (79). The same regional discrepancies were observed after stimulation of total PBMC with three different antigen extracts from *M. tuberculosis*, and the reactivity to these antigens was shown to be at least partially dependent on the presence of IL-12 and IL-18, supposed to be derived from accessory cells.

Conradie et al. (84) described that the level of activation of PB NK cells (frequency of CD69^+^ and CD69^+^HLA-DR^+^ events) allowed, among other parameters, to discriminate between *M. tuberculosis*-induced immune reconstitution syndrome, HIV infection and co-infection with both pathogens.

Although these three papers suggest some influence of *M. tuberculosis* on PB NK cells, it is not clear yet to which extent NK cells really intervene in the immune defense against this pathogen that persists in the lungs. An investigation on tuberculous pleurisy (85) revealed a large predominance of CD56^bright^CD16^–^ NK cells in the pleural fluid, and an apoptotic effect of soluble factors from this environment predominantly on CD16^+^ NK cells. *M. tuberculosis* induced IFN-γ production from CD56^bright^ NK cells in the absence of monocytes, T cells and B cells, leaving open the possibility of a direct productive interaction between the bacteria and the NK cells.

Lai et al. (86) presented a work on nontuberculous mycobacterial lung infections, which means due to other mycobacterial species, such as *Mycobacterium abscessus* and *Mycobacterium kansasii*. As the latter become more and more prevalent in developed countries, these authors performed a study in C57BL/6 mice that were infected intratracheally with *M. kansasii*. They found that NK cell depletion increased bacterial burden, mortality, and pathogenetic postinfectious changes (macrophage phagocytosis, DC activation, cytokine production, and development of granuloma). The same observations were made in IFN-γ-KO animals and restored after transfer of wildtype NK cells. These cells were also the most important producer of IFN-γ in this model (86). Lai et al. further cited papers that had demonstrated a similar protective effect of IFN-γ produced by NK cells in the infections with *Bordetella pertussis*, *Francisella tularensis*, and *Chlamydia muridarum* in mouse models of respiratory infection.

Previous publications by the same group had shown that NK cells can directly lyse *M. tuberculosis* and *M. kansasii* via the cytotoxic proteins granulysin and perforin in a contact-dependent manner disrupting mycobacteria cell wall integrity (87), and that in some patients with mycobacterial infections, anti-IFN-γ autoantibodies were detected (88). The killing process involved signaling through NKG2D and NCR as well as MAP kinases, suggesting that similar mechanisms are involved for the killing of bacteria and of eukaryotic target cells (87). This is potentially a very important observation, as it strongly suggests that both conventional cytotoxic mechanisms and cytokine production might be relevant in anti-mycobacterial defense.

Some studies were also performed on *Mycobacterium bovis* Bacillus Calmette-Guérin (BCG), an attenuated mycobacterial strain used as an anti-tuberculous vaccine (89). For example, it was demonstrated *in vitro* that CD56^bright^ NK cells reacted to this microorganism by proliferation and IFN-γ production, whereas their CD56^dim^ counterparts better up-regulated the cytolytic proteins perforin and granzyme A (90), all of which was largely expected based on what is known about the functional specialization of these two NK cell subsets (1–3). In a mouse *in vivo* model, where BCG was directly administered (intratracheally) into the lungs, NK cell-mediated production of IFN-γ rapidly increased in the first days after infection, similarly to the number of lung NK cells (89). After NK cell depletion, the reduction of body weight was less pronounced compared to non-depleted mice, whereas the bacterial load remained identical. Importantly, inflammation and injury of the pulmonary structures was much less pronounced in the NK cell-depleted animals, suggesting a pathogenic role for these lymphocytes. Indeed, the level of pro-inflammatory cytokines and chemokines was also reduced in the absence of NK cells, and the percentages of IFN-γ^+^CD4^+^ and IFN-γ^+^CD8^+^ T cells was significantly increased in these mice. Bacillus Calmette-Guérin-infected macrophages up-regulated NKG2D ligands, which induced their lysis via this receptor-ligand interaction. Finally, the blocking of NKG2D with a monoclonal antibody restored the survival of the macrophages and the T cell-mediated immune response (89).

It is difficult to make a coherent synthesis of all these observations on NK cells and mycobacteria, but it is nevertheless quite appealing that again positive, negative and neutral aspects are described, which may vary according to the models and the experimental setup. This shows that NK cells still hide a lot of secrets regarding their function in anti-mycobacterial infections as well as in bacterial pathogenesis overall. A summary of the relationships between the bacteria discussed and NK cells is presented in Table 2.

**TABLE 2:** Natural Killer (NK) cells and different bacteria.

Bacteria	Sp. infected	Origin of NK cells	Effects on/of NK cells	References
*Pseudomonas aeruginosa*	Mouse	Lung	↑ Clearance via NKG2D	(51)
*P. aeruginosa* exotoxin A	Human	Peripheral blood	↓ Cytotoxicity (K562)	(53)
*Burkholderia cenocepacia*	Human	Peripheral blood	Killing of bacteria	(55)
*Klebsiella pneumoniae*	Human	Peripheral blood	↑ IFN-γ, ↑α-defensin	(59)
*Klebsiella pneumoniae*	Mouse	Lung	↑ IFN-γ, ↑ IL-22	(60)
*Legionella pneumophila*	Mouse	Lung, spleen	↑ cytotoxicity, ↑ IFN-γ	(67)
*Streptococcus pneumoniae*	Mouse	Lung	↑ IFN-γ, ↑ IL-10	(71)
*Staphylococcus aureus*	Mouse	Airways	↑ Number/activation	(76)
*Mycobacterium tuberculosis*	Mouse	Lung	↑ Number, ↑ IFN-γ	(81)
*Mycobacterium tuberculosis*	Human	Peripheral blood	↑ IFN-γ	(23)
*Mycobacterium kansasii*	Mouse	Lung	↑IFN-γ	(86)
Mycobacteria	Human	Peripheral blood	Killing of bacteria	(87)
*Mycobacterium bovis* BCG	Mouse	Lung	↑ IFN-γ, ↑ number	(89)
*Mycobacterium bovis* BCG	Human	Peripheral blood	↑ IFN-γ, ↑ PF, ↑ GZM A	(90)

*Summary of the effects of and/or on NK cells in infections with the listed bacterial pathogens. Sp., species; IFN-γ, interferon-gamma; BCG, bacillus Calmette-Guérin; IL, interleukin; PF, perforin; and GZM A, granzyme A.*

## Chlamydia

These are obligate intracellular pathogenic bacteria that are responsible for several types of human and mouse diseases. Various studies dedicated to this type of microorganisms illustrated the concept that NK cells usually do not respond as a pure population as may be the case for *in vitro* experiments, but that *in vivo* they are part of a tightly controlled immune network composed of cells, cytokines, chemokines and exosomes.

Thus, in mouse models, NK cells influenced the interaction between DC, T helper (h)1 and Th17 T lymphocytes in *C. muridarum* lung infection (91), modulated the balance between Th1 and Th17 T cells and T regulatory cells (Treg) in the same type of infection (92), and again positively regulated the interactions between DC and T lymphocytes against *Chlamydophila pneumoniae* (93). In all these situations, NK cells exerted a protective and disease-controlling effect via their influence on the bridge between innate and adaptive immune responses.

With the ambitious aim to experimentally investigate the famous “hygiene hypothesis,” Han et al. (94) studied mice infected with *C. muridarum* and rendered allergic to ovalbumin (OVA). They observed that prior infection could inhibit at least certain parameters of allergy. However, NK cell depletion partly suppressed the “beneficial” effect of the lung infection. Adoptive transfer of NK cells from infected mice inhibited partially the development of an allergic response in non-infected recipients. NK cell-devoid mice coherently produced more Th2 type cytokines (“pro-allergic” Th2 cytokines, IL-4, and IL-5) than IFN-γ (“anti-allergic” Th1 cytokine).

A detrimental effect of NK cells had been shown for the immune response to the respiratory rodent pathogen *Mycoplasma pulmonis*, related to the human infectious agent *Mycoplasma pneumoniae*. Indeed, in a quite complicated experimental setup, Bodhankar et al. demonstrated that NK cell depletion interfered positively with the development of a protective adaptive immunity after nasal-pulmonary immunization with bacterial antigens (95). This could be explained because NK cells shaped the T cell cytokine response toward more IL-4, IL-13, and IL-17 but away from IFN-γ production.

## Natural Killer Cells as Clinical Indicators in Respiratory Infectious Diseases in Children

Wurzel et al. presented large cohort studies of children with protracted bacterial bronchitis (PBB) and mild bronchiectasis, associated or not with human adenovirus co-infection (96, 97). Besides typical socio-economic and clinical factors, an elevated NK cell number relative to the values of healthy children of the same age was observed in the PB of diseased children in general and with adenovirus species C particularly. NK cell phenotype and function were not further investigated.

## A Human Kir Recognizes a Conserved Bacterial Epitope

Recently, Sim et al. (98) made the important discovery that the HLA-C-specific activating KIR2DS4 did recognize a conserved bacterial peptide presented by HLA-C, and more precisely by HLA-C^∗^05:01. The sequence of the peptide required for this recognition was a “rare” self-peptide, but the epitope of interest is conserved in the recombinase A (RecA) of many bacterial species (more than 1000 according to the authors’ claims), most of them belonging to serious human pathogens, such as *Helicobacter pylori*, Brucella, *Campylobacter jejuni*, and *Chlamydia trachomatis* (98). Interestingly, activation of resting NK cells via KIR2DS4 alone was sufficient to induce degranulation and cytokine production, whereas all other known AR, except CD16, need at least one co-activating molecule engaged at the same time (99). There was, furthermore, an inverse correlation between the frequency of the KIR2DS4 full length gene and the HLA-C^∗^05:01 allele. Thus, it appears that the KIR family is not only involved in NK cell licensing and in multiple disease associations, but also, most likely, in antibacterial defense. This paper received an accompanying Commentary by Peter Parham, which places the findings in the broader context of KIR and HLA class I molecules (100).

To sum up, NK cells might be directly activated by various bacteria via contact-dependent mechanisms whose modes of functioning are still unknown, via TLR, via KIR2DS4, or more indirectly via the up-regulation of ligands for their AR, such as NKG2D, by infected cells. They might also react to cytokines released into the microenvironment by antigen-presenting cells (macrophages, DC).

## State-Of-The-Art Methods for Investigation of the Lungs

Dietert et al. published a plea for the histopathological evaluation of the consequences of different infectious lung diseases in mouse models and described the pathogen-specific features characteristic for each of them (101). Indeed, many variations were observed between the infecting microorganisms, be they bacterial or viral in nature. The authors emphasized that histopathology remains the “most conclusive and practical read out” for the evaluation of the effects of the various infectious models on mouse lungs.

Although this is true, more “modern” and state-of-the-art methods are being developed and are about to enter the laboratories, as a consequence of general scientific and technical progress but also of the “3R” approach regarding experiments with animals.

In 2019, the team of Hans Clevers described the generation of human airway organoids derived from surgical material or from BALF (102). These were long-term proliferating structures that recapitulated a normal airway with different types of cells that are physiologically present *in vivo*. The beauty of the system *per se* was already an accomplishment, but it could be used for the study of various lung diseases, such as cystic fibrosis, cancer, or viral infections (102). Therefore, it is likely that bacterial infections could similarly be investigated in this system, and the data obtained would probably be more relevant to human pathology than the mere mouse models (and save the life of many mice by the way).

The same year, Ross et al. (103) published a review on the “*ex vivo* human lung.” They worked with donor lungs not retained for transplantation, extracted primary cells from them and developed an “*ex vivo*-perfused single human lung” that would allow the investigation of different lung diseases. The system seems at first sight less elegant than the lung organoids and is maybe also more limited in the spectrum of possible pathologies that can be investigated. The advantage would be that an entire, complete organ is available and not just an organoid.

Yet another option is the “alveolus-on-a-chip,” developed by Deinhardt-Emmer et al. (104). It was a three-dimensional structure with an air phase and a liquid phase, where endothelial cells, epithelial cells and macrophages could be co-cultured. In the presented work, a primary influenza virus infection, followed by a *S. aureus* superinfection, were investigated, and it was shown that the endothelium was seriously damaged under these conditions.

Likewise, single cell transcriptomics is a powerful tool that can reveal huge amounts of details about all kinds of immune cells, and among them NK cells, as exemplified by lung cancer-infiltrating immunocytes in human and mouse (105).

## Natural Killer Cells as a Therapeutic Option for Airway Infections? Cave Canem!

One aspect of NK cells is their putative potential for a dual role as “pro-inflammatory” and “regulatory” effectors, which might be mediated by different subsets (106). Our group has previously touched the problem that NK cells are in fact a double-edged sword, meaning that they might have sometimes beneficial but sometimes rather deleterious effects (72). This has again become clear throughout this review, although the models and studies presented and discussed were all but homogeneous. It might be expected that this will change in the coming years if more and more teams will use the organoid and organ on-a-chip technologies and go into various “omics.” Overall, given the current and justified hype for NK cells as efficient agents for cancer immunotherapy, it would be difficult to convince the NK community that their favorite cells might also have a dark side. We emphasize that several methods to improve NK cell antitumoral efficiency, such as particularly CAR-NK cells (10) and NK cell engagers, recently described by the Vivier group (107), should be sufficient to stand up for the use of NK cells in this indication.

However, what about the therapeutic indication of NK cells in infectious diseases in general and in the lungs particularly? Due to the current COVID-19 pandemic, this question has gained increased interest (108, 109), and in addition, most of the papers discussed here that describe an influence of NK cells on the disease course in the airways conclude with the statement that NK cells should be targeted in respiratory infections. But it has clearly been shown that these lymphocytes can have detrimental side effects and cause significant damage to the airways, at least in viral diseases (108, 109). Available literature does not give clear indications regarding bacterial pathogenesis, but the issue was already discussed in 2012, with the question if NK cells are angels or devils in bacterial infectious diseases (50). This problem is, in our opinion, not yet resolved and a lot of research work will be necessary in the field, keeping in mind that the number of multi-resistant bacterial strains is increasing at a terrifying rate and that alternatives to antibiotics must be discovered and developed.

Finally, a general problem in the field and a caveat to many of the presented studies is the difficulty of distinguishing NK cells reliably form ILC1, which also produce IFN-γ as a signature cytokine and have a partially overlapping phenotype. A high plasticity within the ILC family renders even possible the conversion of NK cells, in certain microenvironments, into ILC1-like cells (110–112). However, whereas both NK cells and ILC1 require the transcription factor T-bet for their development and function, NK cells need and express Eomes in addition. ILC1 are preferentially located in tissues and are very rare in peripheral blood, in contrast to NK cells. Thus, one can be confident that the studies discussed here that worked with blood (human) and blood or spleen (mouse) NK cells really investigated NK cells and not ILC1. For tissue-based studies, the differences might be more blunted, although ILC1 are considered as non-cytotoxic cells (110–112). These difficulties are in line with the increasing number of “new” cell types that are currently discovered [for example MR1 T cells (113)], as a consequence of the ever growing diversification and performance of the experimental tools in immunology.

## Author Contributions

MT and NP conceived the article, participated to bibliographic research, and critically reviewed the final revised manuscript. JZ conceived the article and wrote the manuscript. All authors contributed to the article and approved the submitted version.

## Conflict of Interest

The authors declare that the research was conducted in the absence of any commercial or financial relationships that could be construed as a potential conflict of interest.
